# Prevalence, patterns and motives for hormonal contraceptive use among German servicewomen: results from a nationwide survey

**DOI:** 10.3389/fendo.2026.1844924

**Published:** 2026-06-19

**Authors:** Jennifer Schlie, Timo Schinköthe, Annette Schmidt

**Affiliations:** 1 Institute for Sports Science, Chair of Sports Biology, University of the Bundeswehr Munich, Munich, Germany; 2 Kompetenzzentrum für Funktionsfitness, University of the Bundeswehr Munich, Neubiberg, Germany

**Keywords:** combined oral contraceptives, estrogen, female soldiers, German armed forces, hormonal contraception, long-acting reversible contraceptives, progestin-only contraceptives, progestogen

## Abstract

Hormonal contraceptive (HC) use is common among servicewomen and has been discussed in relation to health, performance, and operational readiness. No systematic data exist on HC prevalence among German servicewomen. This study analyzes contraceptive practices and motivations for contraceptive choice in German servicewomen. A nationwide, cross-sectional online survey was conducted among female members of the German Armed Forces. Recruitment occurred via military institutions and a digital flyer. The anonymous questionnaire (40 items, 10–15 min) assessed demographic characteristics and contraceptive use. Data were cleaned and analyzed using R. Inferential analyses comprised pairwise adjacent comparisons using Fisher’s exact test with Holm correction. A total of 2818 servicewomen completed the survey (response rate 11.2%; mean age 33.9 years). Overall, 38.1% reported current HC use. Combined oral contraceptive pills (COCP) were the most common method (18.5%), followed by hormonal intrauterine devices (8.9%) and progestin-only pills (8.4%). HC use declined with increasing age, primarily due to decreasing pill use, although 8% of women ≥40 years still reported COCP use. Systemic long-acting reversible contraceptives (LARC) (implants and injections) were virtually absent. Contraceptive use differed significantly across age- and length-of-service categories, but not BMI categories. Pregnancy prevention was the primary reason for HC use (82%), followed by management of menstrual pain and bleeding. A small proportion reported HC use for performance-related cycle control (13%) or masking of amenorrhea (2%). Deployment-related switching to HC was uncommon. HC use among German servicewomen appears low compared with reports from British and U.S. militaries. Given evidence linking certain systemic LARC formulations to potential effects on bone metabolism, the low prevalence warrants further investigation in the context on musculoskeletal health and injury risk in a population exposed to high mechanical loading. Preventive efforts in the German military should consider a broader range of modifiable risk factors, including nutrition and training load. The findings may indicate potential gaps in military-specific education and counseling regarding informed contraceptive choice, occupational implications of different methods, and age-related hormonal management. The response rate and the limited scope of certain questionnaire items should be considered when interpreting the results.

## 1 Introduction

**Figure 1:**
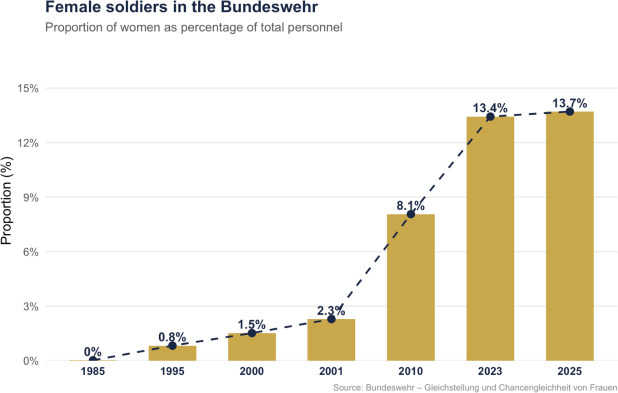
Promotion of female soldiers in the German Armed Forces over time. Image visually adapted from publicly available information provided by the Bundeswehr (1).

Over recent decades, the participation of women in military forces worldwide has steadily increased as policies have expanded roles and opportunities for female service members. In the German Armed Forces (Bundeswehr), women currently comprise approximately 13% of total personnel. This corresponds to about 25,200 female soldiers serving across branches and functions – a marked increase from 6,731 in 2001, when women gained access to all military professions in Germany (**Figure 1**) (1). Female soldiers may demonstrate certain physiological advantages over their male counterparts under specific conditions, including better performance under psychological and metabolic stress (2), as well as ergonomic advantages due to their smaller body size (3). Their increasing integration into military roles has been associated with growing attention to sex-specific health outcomes. For instance, a substantial body of international research reports substantially higher rates of musculoskeletal and bone stress injuries among female soldiers, with relative risks often three- to six-fold higher than in men (4–7). These findings highlight the importance of further investigating female-specific physiological determinants within military populations (8). Sex differences in injury incidence have been attributed, in part, to biomechanical and anthropometric factors, such as lower muscle mass and smaller body size relative to standardized external load (3). Increasingly, however, endocrine factors are recognized as potential contributors to distinct injury patterns in women (9–11). Estrogen is a key regulator of bone turnover and skeletal adaptation in women (12), and endogenous estrogen levels may be influenced by various factors including energy availability (13) but also lifestyle factors such as hormonal contraceptive (HC) use. Among these factors, HC use represents a particularly relevant and modifiable endocrine exposure in women of reproductive age. While contraceptive methods differ substantially in their endocrine effects, some systemically acting progestin-only methods may suppress endogenous estrogen production and have therefore been associated with unfavorable effects on bone metabolism, particularly when used before peak bone mass is fully attained (14).

### 1.1 Classification of contraceptive methods

HC methods vary substantially with respect to hormonal composition, systemic exposure, and suppression of ovulation (**Table 1**). From a biomedical perspective, contraceptives can be classified into three major categories. Non-hormonal methods include copper intrauterine systems, barrier methods (e.g., condoms, diaphragms), and fertility awareness methods. They act without systemic hormonal modulation but may pose limitations in military settings due to variable contraceptive efficacy, limited protection of sexually transmitted infections (15), and preservation of the natural menstrual cycle, which may increase logistical and symptomatic burdens in field environments. Combined HC, including combined oral contraceptive pills (COCPs), vaginal rings, and transdermal patches, contain both estrogen and progestin. They primarily prevent pregnancy by suppressing ovulation, stabilizing endometrial growth, and thickening cervical mucus (16). Their systemic hormonal effects often result in more predictable bleeding patterns and allow intentional menstruation suppression, which may be advantageous in physically demanding contexts. Progestin-only contraceptives (POCs) comprise progestin-only pills (POP) implants, injections of depot medroxyprogesterone acetate (DMPA), and hormonal intrauterine systems (IUS). These methods differ substantially in their duration of action and systemic hormonal exposure (**Table 1**). POPs are classified as short-acting reversible contraceptives (SARC), and are taken on a daily basis. They primarily act through changes in cervical mucus, with variable suppression of ovulation. In contrast, implants, DMPA and hormonal IUS are classified as long-acting reversible contraceptives (LARCs). While implants and DMPA provide prolonged systemic progestin exposure and frequently induce amenorrhea, hormonal IUSs (e.g., levonorgestrel-releasing systems) are LARCs that act predominantly locally with minimal systemic hormone levels (15). Throughout this manuscript, the term hormonal contraception (HC) refers to all estrogen- and/or progestin-containing contraceptive methods.

**Table 1:** Overview of hormonal contraceptive methods by composition, systemic effects and route of administration.

Category	Subtype, examples	Hormonal composition	Mechanism, (suppression of ovulation?)	Systemic hormonal exposure	Route of administration
*Combined oral contraceptive pill (COCP)*	Monophasic, biphasic, triphasic pills	Ethinylestradiol or estradiol + progestin (e.g. levonorgestrel)	Yes (consistent ovulation suppression via HPO-axis inhibition)	Yes, exogenous estrogen and progestin with daily pharmacokinetic fluctuations	Oral (daily)
*Combined non-oral methods*	Vaginal ring, transdermal patch	Ethinylestradiol + progestin	Yes (consistent ovulation suppression)	Yes, constant systemic estrogen and progestin exposure	Vaginal/transdermal
*Progestin-only contraceptives (POC)*	Short-acting reversible contraception (SARC)				
	Progestin-only pill (“minipill”)	Progestin (e.g. desogestrel, levonorgestrel)	Variable (complete with desogestrel; inconsistent with others)	Yes, systemic progestin exposure, no estrogen	Oral (daily)
*Progestin-only contraceptives (POC)*	Long-acting reversible contraception (LARC)				
	Implant	Etonogestrel	Yes (highly reliable ovulation suppression)	Yes, continuous systemic progestin levels, no estrogen	Subdermal
	DMPA	Medroxyprogesterone acetate	Yes (highly reliable ovulation suppression)	Yes, continuous systemic progestin levels, no estrogen	Intramuscular or subcutaneous (12-week interval)
	Hormonal intrauterine system	Levonorgestrel	No (ovulation usually preserved)	Minimal systemic progestin exposure, no estrogen	Intrauterine

HPA, hypothalamic-ovarian-axis; DMPA, depot Medroxyprogesterone acetate.

Each contraceptive class may exert distinct endocrine effects that may interact with bone turnover, energy availability, and training adaptation. These interactions may be relevant to bone health and physical performance, although their magnitude and clinical significance likely depend on the specific contraceptive and population studied. Given the young age at which many women enter the military and the substantial physical demands associated with military service, this population may provide a particularly relevant setting in which to investigate the potential effects of different contraceptive methods (17).

### 1.2 Contraceptive use in international armed forces and elite sports

The modern soldier is often conceptualized as a “tactical athlete”, exposed to training and operational demands comparable to elite sport (18). In athletic populations, HC use is common (19, 20) and driven by multifactorial considerations extending beyond pregnancy prevention (21). In Denmark, 57% of elite female athletes use HC (22), while recent studies in German athletes reported prevalences ranging from 29% to 42% (23, 24). This variability likely reflects differences in study populations and sports disciplines, as one study included athletes from a broad range of sports, whereas the other focused specifically on elite female team sports. Given similar physical demands and operational constraints faced by servicewomen and athletes, especially during training phases and deployments, it may be that similar factors influence contraceptive choices among servicewomen. Since women usually enter the military at a young age and remain in service throughout their reproductive years, contraceptive use is likely relevant for a substantial proportion of servicewomen.

Several studies in military cohorts have quantified the prevalence of and reasons for contraceptive use. Recently, the British military published data on self-reported HC use among British servicewomen, reporting an overall prevalence of 58%, with predominant use of COCPs and IUSs (20% and 17%, respectively). An additional 13% used systemic LARCs, including implants and DMPA injections (20). The study highlighted limitations of military health data systems, particularly under-ascertainment when prescriptions occur outside military health services. Similarly, data from the US military reported that 45.8% of servicewomen used HC in 2021. These observations may however also underestimate, as they may not reflect prescriptions made outside of military health services (25). Beyond prevalence, related research in British military populations has linked menstrual function, energy availability (26, 27), and bone health outcomes such as stress fracture risk and bone turnover markers (10, 11), indicating complex interactions between endocrine factors and injury risk in servicewomen.

Given the high physical demands and the high prevalence of HC use reported in other international armed forces, it remains unclear whether female soldiers in Germany show similar patterns. To date, no study has systematically assessed the prevalence, patterns and motivations of HC use among German servicewomen. In particular it remains unclear, whether HC use in this population reflects patterns observed in other military cohorts or is shaped by country-specific healthcare structures and cultural factors. Differences in the availability of structured education on women’s health and contraception – well established in the British military (28) but limited in Germany – may influence knowledge and decision-making processes, potentially resulting in different contraceptive use patterns between countries.

### 1.3 Rationale and aims of the study

To date, no systematic data exist on contraceptive use among female servicemembers in the German Armed Forces. Establishing robust data on contraceptive practices is a necessary first step toward understanding reproductive health and informing future research on potential health and performance implications in female soldiers. The present study therefore aimed to characterize self-reported HC use and underlying motivations among German servicewomen and to examine whether contraceptive choices vary in the context of anticipated deployments. We hypothesized that a) the prevalence of HC would be comparable to that reported in other European armed forces, and b) that occupational factors would play a major role in contraceptive decision-making beyond pregnancy prevention.

## 2 Materials and methods

### 2.1 Study design and recruitment

This study was conducted as an online survey between October 2025 and February 2026. Eligible participants were a) female and b) regular members of the German Armed Forces. There were no explicit age or length of service restrictions. The questionnaire was generated and administered via SoSci Survey (version 3.7.06 (1)), available to users via www.soscisurvey.de. The survey aimed to reach all female servicemembers of the German Armed Forces. Recruitment was facilitated through a digital flyer with a participation link and QR code that was distributed to all military bases, military hospitals, and military universities across Germany via the occupational health management coordinators. In addition, the flyer was disseminated through social media channels and displayed in publicly accessible locations such as sports facilities, cafeterias, and canteens. Participation was voluntary and unpaid; however, the survey was promoted as anonymous, time-efficient, and contributory to improving health and operational readiness among female servicemembers. As participation was voluntary and based on an open online survey, a self-selection of participants cannot be ruled out.

The study was conducted in accordance with the Declaration of Helsinki and received ethical approval from the Ethics Committee of the University of the German Armed Forces Munich, Germany (EK UniBw M 25-32). Approval to conduct the survey was also granted by the Federal Ministry of Defense (3/5/25). Prior to participation, all respondents were provided with detailed information regarding the study aims, data protection, and participation procedures. Electronic informed consent was required to access the survey. Upon completion of the questionnaire, participants were asked to confirm submission of their responses by selecting “Submit”; alternatively, they could close the browser window to ensure that their responses were not recorded.

### 2.2 Questionnaire

The questionnaire was specifically developed for this study and comprised 40 items, with an estimated completion time of 10–15 minutes. The number of items presented varied depending on individual response pathways. The questionnaire was developed by the study team based on the research objectives and existing instruments. Selected items were adapted from previously validated questionnaires including the Low Energy Availability in Females Questionnaire (LEAF-Q) (29) and the Bewegungs- und Sportaktivitätsfragebogen (BSA), a validated instrument commonly used in German-speaking rehabilitation, behavioral, and sports psychology research (30). Items adapted from the LEAF-Q and BSA retained their original content where possible or were modified where necessary to reflect the military context. Additional items were developed to address military-specific aspects such as deployment-related contraceptive use. Prior to the main survey, the questionnaire underwent feasibility and comprehensibility testing in a sample of female servicemembers participating in a separate ongoing research project at our institution. No formal content validation of the newly developed items was performed. The complete questionnaire is available in the open science framework (OSF) repository associated with this study. Most items were presented in a multiple-choice format. A limited number of questions included short free-text response fields, such as those assessing body height and body mass. The present analysis focuses exclusively on a subset of the data collected as part of a larger survey on physical activity, military fitness, menstrual cycle function, HC use, musculoskeletal injury, and lifestyle factors. Specifically, this publication addresses demographic characteristics and contraceptive use. Participants were asked whether they currently used hormonal contraception and, if applicable, to specify the contraceptive method (e.g. COCP, progestin-only pill, hormonal IUS, implant, vaginal ring, depot injection, or other methods), duration of use, reasons for use, and whether occupational factors or anticipated deployments influenced contraceptive choice. Reasons for use were assessed using predefined response categories adapted from the LEAF-Q (29), with multiple responses permitted. The questionnaire did not collect any directly identifiable personal information, and participants could select “I do not know” or “I prefer not to answer” for all items.

### 2.3 Statistical analysis

As this study was designed as an exploratory nationwide survey targeting the entire population of female service members, no *a priori* sample size calculation was performed. All data were downloaded from the SoSci.survey.com platform in Excel format. Data were cleaned prior to analysis, and all free-text responses were screened for potential identifying information, which, if applicable, was removed. Values coded as -9 were recoded to NA and excluded from all analyses (complete-case analysis). Denominators (*N*) reflect the number of participants with valid responses for each variable and are reported in the respective table sections. No imputation was performed.

Descriptive statistics were used to summarize demographic characteristics and the distribution of contraceptive methods. To examine whether the distribution of current HC use differed across demographic subgroups, inferential analyses were conducted. Demographic variables (age, length of service, BMI) are ordered categorical predictors. To detect monotonic trends while controlling the Type I error rate, adjacent subgroup comparisons were applied. Each group was tested only against the immediately consecutive group. This approach avoids the inflation associated with all pairwise combinations and is standard practice for ordered predictors. Contraceptive method was treated as a multinomial outcome variable. For each adjacent subgroup comparison, contingency tables were analyzed using Pearson’s chi-square test when all expected cell counts were ≥5. When this assumption was violated, Fisher’s exact test was applied. For larger contingency tables with sparse cell counts, Fisher’s exact test with Monte Carlo simulation was used to obtain reliable p-values. Comparisons were not performed when empty margins preluded statistical testing. Proportion estimates were accompanied by 95% confidence intervals (Wilson score interval). Cramér’s *V* is reported as an effect-size measure alongside χ²- and Fisher *p*-values; conventional benchmarks are *V* ≈ 0.10 (small), *V* ≈ 0.30 (medium), and *V* ≈ 0.50 (large) (31). All analyses were conducted using R (version 4.5.2). To account for multiple testing within each variable (e.g., age categories, length of service), p-values were adjusted using the Holm correction. Statistical significance was defined as adjusted *p* < 0.05.

## 3 Results

### 3.1 Participants and demographics

**Figure 2:**
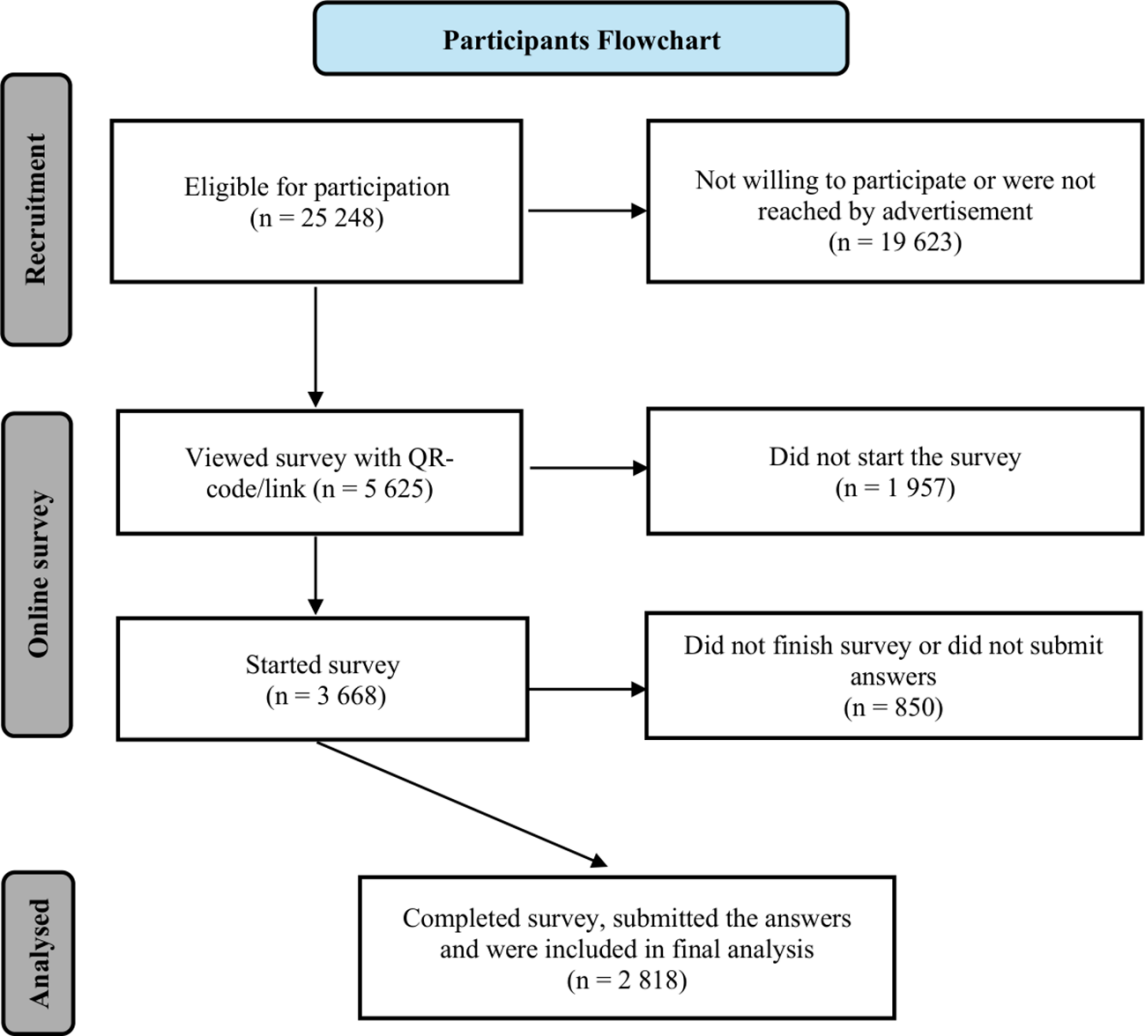
Participants flow through the study. Shown are the number of views, surveys completed and submitted (as a fraction of all 25 248 servicewomen eligible for participation), as well as the exclusions made before analysis.

Across the German Armed Forces, approximately 25,248 servicewomen were eligible for participation in this study (1). While 3,668 women started the digital survey, 2,818 (77%) completed all questions and agreed to submit their answers. The flow of participants is visualized in **Figure 2**. While the response rate of 11.2% was relatively low, the sample included participants from all major service branches, suggesting broad coverage across the German Armed Forces. The largest proportion was serving in the Sanitary Service (33%), followed by the Air Force (22%). Demographic data are shown in **Table 2**.

**Table 2:** Participants characteristics.

Category	*n* (%)
Age (years) (*n* = 2756)	
18–24	430 (16)
25–29	492 (18)
30–34	564 (20)
35–39	570 (21)
≥40	700 (25)
BMI (*n* = 2759)	
<18	14 (1)
18–25	1638 (59)
>25	1107 (40)
Height (cm) (*n* = 2762)	
<160	175 (6)
160–164	593 (21)
165–169	769 (28)
170–174	709 (26)
175–179	352 (13)
≥180	164 (6)
Weight (kg) (*n* = 2727)	
<55	128 (5)
55–64	826 (30)
65–74	909 (33)
75–84	476 (17)
≥85	388 (14)
Service branch (*n* = 2755)	
Army	569 (21)
Air Force	595 (22)
Navy	371 (13)
Cyber and Information Domain	304 (11)
Medical/Support Services	916 (33)
Length of service (years) (*n* = 2686)	
<1	89 (3)
1–4	374 (14)
5–9	620 (23)
10–14	708 (26)
≥15	895 (33)

Demographic data of n = 2818 servicewomen who have responded to the online survey. Data are given as count and percentage [n (%)].

### 3.2 Hormonal contraceptive use

Overall, 38.1% [36.2–40.1%] of servicewomen reported current HC use, while 61.2% reported the use of non-hormonal methods or no contraception (**Table 3**). Percentages for contraceptive methods refer to the total study population unless otherwise stated. COCPs were the most frequently used hormonal method (18.5%). LARC methods were used by 9.7% of servicewomen, of which implants and injectables accounted for only a fraction. 77.5% reported using HC for more than 24 months.

**Table 3:** Hormonal contraceptive method prevalence and length of usage in German servicewomen.

(*n =* 2484)	*n* (%)	95% CI
Currently use a hormonal contraceptive	947 (38.1)	[36.2–40.1%]
Contraceptive type (*n =* 2484)		
Combined oral contraceptive pill	460 (18.5)	[17–20.1%]
Progesterone-only pill	209 (8.4)	[7.4–9.6%]
Implant	11 (0.4)	[0.2–0.8%]
Injection	10 (0.4)	[0.2–0.7%]
Intrauterine system	220 (8.9)	[7.8–10%]
Unsure which method is used	22 (0.9)	[0.6–1.3%]
Length of use (*n =* 947)		
<6 months	53 (5.6)	[4.3–7.2%]
6–12 months	59 (6.2)	[4.9–8.0%]
12–18 months	55 (5.8)	[4.5–7.5%]
18–24 months	46 (4.9)	[3.7–6.4%]
>24 months	734 (77.5)	[74.7–80.1%]
Not reported	0	[4.3–7.2%]
Military profession influences HC-choice (*n =* 947)		
Yes	306 (32.3)	[29.4–35.4%]
No	604 (63.8)	[60.7–66.8%]
Unsure/no reply	37 (3.8)	[2.8–5.3%]

Data are n and percentage as well as 95% CI: Wilson score interval. Percentages for contraceptive methods refer to the total sample of n = 2818 participants. Percentages for duration of hormonal contraceptive use and occupational influence refer only to HC users (n = 947).

### 3.3 Hormonal contraceptive use compared by demographics

Current use of any form of HC varied across demographic subgroups and was most prevalent among women aged 18–24 years (48%) (**Table 4**). Implants and injectables were rarely reported in all age groups. Significant shifts across demographic categories were observed for age and length of service, but not for BMI. Pairwise adjacent comparisons across age categories revealed significant shifts between the age groups 25–29 and 30–34 years (p = 0.004) and between 35–39 and ≥40 years (p = 0.013), whereas no differences in contraceptive distribution were found between adjacent younger or middle age groups. With respect to BMI, adjacent comparisons showed no significant differences in contraceptive distribution either between underweight and normal-weight women or between normal-weight and overweight women. In the analysis stratified by length of service significant shifts were found between women with 1–4 and 5–9 years of service (p = 0.017) and between 5–9 and 10–14 years of service (p < 0.001), while women with 10–14 and ≥15 years of service showed no significant differences.

**Table 4:** Current hormonal contraceptive use in German servicewomen separated by demographics.

	Any type n (%)	COCP n (%)	POP n (%)	Implant n (%)	Injection n (%)	IUS n (%)
Age*						
18–24	207 (48)	127 (30)	41 (10)	2 (0)	1 (0)	28 (7)
25–29	195 (40)	116 (24)	39 (8)	2 (0)	0 (0)	35 (7)
30–34	179 (32)	74 (13)	38 (7)	3 (1)	1 (0)	53 (9)
35–39	180 (32)	86 (15)	39 (7)	1 (0)	4 (1)	45 (8)
≥40	185 (26)	55 (8)	51 (7)	3 (0)	4 (1)	59 (8)
BMI						
<18	4 (29)	1 (7)	0 (0)	0 (0)	0 (0)	3 (21)
18–25	575 (35)	278 (17)	135 (8)	5 (0)	2 (0)	130 (8)
>25	369 (33)	180 (16)	74 (7)	6 (1)	8 (1)	87 (8)
Service branch						
Army	214 (38)	97 (17)	46 (8)	1 (0)	4 (1)	56 (10)
Air Force	211 (35)	102 (17)	47 (8)	2 (0)	2 (0)	48 (8)
Navy	129 (35)	61 (16)	25 (7)	0 (0)	0 (0)	34 (9)
Cyber and Information Domain	94 (31)	53 (17)	16 (5)	2 (1)	3 (1)	18 (6)
Medical/Support Services	301 (33)	147 (16)	75 (8)	6 (1)	1 (0)	64 (7)
Length of service (years)**						
<1	34 (38)	19 (21)	9 (10)	0 (0)	0 (0)	5 (6)
1–4	164 (44)	97 (26)	41 (11)	0 (0)	1 (0)	19 (5)
5–9	197 (38)	118 (23)	26 (5)	4 (1)	2 (0)	35 (7)
10–14	160 (30)	64 (12)	49 (9)	1 (0)	1 (0)	41 (8)
≥15	259 (29)	98 (11)	59 (7)	3 (0)	3 (0)	84 (9)

BMI, body mass index; COCP, combined oral contraceptive pills; POP, progestin-only pills; IUS, intrauterine system. Inferential statistics comprised pairwise adjacent comparisons using the Fisher’s exact test (with/without Monte Carlo simulation).

*Significant differences in contraceptive distribution were observed between age groups: 25–29 vs 30-34 (p = 0.004); 35–39 vs ≥40 (p = 0.013).

** Significant differences in contraceptive distribution were observed between length of service groups: 1–4 vs 5-9 (p = 0.017); 5–9 vs 10-14 (p < 0.001).

Across service branches, the proportion of HC use ranged from 33% in the Cyber and Information domain to 41% in the army. The differences between branches were however not significant (**Appendix Table A2**).

### 3.4 Reasons for hormonal contraceptive use

The most common reason for HC use was contraception (83%), followed by reduction of menstrual pain (48%) and reduction of menstrual bleeding (41%) (**Table 5**). Performance-related cycle control was reported by a small proportion (13%), as was the masking of amenorrhea (2%). Overall, 32.3% agreed that their military occupation influenced their contraceptive choice (**Table 3**). When stratified by contraceptive method, differences in reported reasons for use were observed. While pregnancy prevention was the predominant reason across all methods, cycle-related indications such as reduction of menstrual bleeding were more frequently reported among users of POP methods and IUS, whereas performance-related reasons were more commonly reported among COCP users.

**Table 5:** Reasons for hormonal contraceptive use among German servicewomen.

Category	Prevent pregnancy n (%)	Reduce menstrual pain n (%)	Reduce/stop bleeding n (%)	Performance n (%)	Mask absent periods n (%)
Any (*n* = 910)	756 (83)	433 (48)	370 (41)	117 (13)	19 (2)
COCP (*n* = 460)	390 (85)	222 (48)	148 (32)	75 (16)	15 (3)
POP (*n* = 209)	162 (78)	108 (52)	97 (46)	28 (13)	3 (1)
Implant (*n* = 11)	10 (91)	5 (45)	7 (64)	4 (36)	0 (0)
Injection (*n* = 10)	8 (80)	4 (40)	6 (60)	0 (0)	0 (0)
IUS (*n* = 220)	186 (85)	94 (43)	112 (51)	10 (5)	1 (0)

Data are n and percentage and relative to the proportion of servicewomen who reported using hormonal contraceptives (n = 947). This item was adapted from the official LEAF-Q diagnostic tool (29). Multiple answers were allowed for this item.

### 3.5 Hormonal contraception in the face of deployments

Ten percent of servicewomen reported having temporarily switched to a (different) HC during a previous deployment or similar assignment, while the majority indicated no such change (**Table 6**). Eleven percent of respondents indicated plans to temporarily switch to a (different) HC in future deployments. One third (29%) reported having consciously used HC during a deployment to suppress or postpone menstruation, whereas 22% of respondents indicated plans to do so in future deployments. Among servicewomen who reported having switched to hormonal contraception during a deployment, the majority opted for short-acting methods such as COCP (39%), whereas 12% selected a systemic long-acting method (implant or DMPA) (**Appendix Table A1**). Similarly, among those planning to use HC in future deployments, 8% indicated an intention to use an implant or DMPA. Due to the exploratory nature of these items, results are presented descriptively without inferential statistical testing.

**Table 6:** Hormonal contraceptive use during deployments.

(*n* = 2818)	*n* (%)
Have changed HC method during deployment/operations	273 (10)
Have intentionally used HC to suppress periods during deployment	823 (29)
Plan to change HC method for future deployment	302 (11)
Plan to use HC to suppress periods in future deployment	607 (22)

Data are n and percentage and relative to the total sample of n = 2818 servicewomen.

## 4 Discussion

The increasing proportion of women in military forces worldwide has highlighted the persistent gender data gap in military health research. A better understanding of female-specific reproductive health and its potential relevance for performance, readiness, well-being, and injury prevention (20) is therefore essential to inform gender-sensitive training, medical care, and operational planning. Importantly, such knowledge may help shift the focus away from perceived physiological disadvantages toward leveraging female-specific physiological capabilities (2). As a first step in addressing this gap, the present study examined the prevalence, patterns, and reasons for HC use among German servicewomen. Contrary to expectations, HC prevalence was moderate at 38.1%, with approximately 61% reporting non-hormonal- or no contraception. COCP were the most frequently used method (18.5%), followed by the IUS (8.9%).

### 4.1 Prevalence of HC use

Comparisons with the general German population are limited due to differences in the categorization of contraceptive methods. While around 31% of German adults use pills and approximately 15% IUS, there is no differentiation between combined or progestin-only formulations or hormonal versus copper intrauterine systems (32, 33). Against this background, HC use among German servicewomen does not appear markedly elevated. Notably, the proportion of pill use (COCP: 18.5%; POP: 8.4%) was even lower than in the general population, and overall prevalence of HC falls within the range reported for German elite athletes (23, 34). These moderate numbers of HC use in Germany may reflect a broader societal trend toward a more critical attitude toward hormonal contraception, driven by concerns about potential physical and psychological side effects (32). Similar patterns have been reported among German athletes, who appear to use HC less frequently than athletes in other European countries such as Denmark (22) and Norway (35). Overall, the observed contraceptive patterns may reflect influences beyond medical considerations and performance optimization alone, including cultural attitudes and risk perceptions.

International comparisons illustrate variability of contraceptive patterns across military populations. Myers et al. reported a HC prevalence of 58% among British, with COCP being the most commonly used method (20). Further, systemic progestin-only methods such as implants and injections were common in the UK (13%) but were virtually absent in the present German cohort. Data from the United States likewise indicate a substantially higher uptake of HC, with LARC methods accounting for approximately 22.4% and SARC representing about 23.4% (25). A study conducted among servicewomen in the Democratic Republic of Congo reported a prevalence of only 24.9% for any form of modern contraception (36). Such findings highlight the considerable global variability in contraceptive patterns, likely reflecting differences in healthcare infrastructure, availability of contraceptive methods, sociocultural norms, and reproductive health education.

The majority of HC users (78%) reported use for more than 24 months, which is comparable to observations in other military populations (20). This may indicate that HCs in military populations are typically used over extended periods, which may be associated with early initiation, stable occupational conditions, and a preference for reliable methods compatible with military training and operational demands.

### 4.2 HC use by demographics

Contraceptive use varied across age groups and career stages, with a general decline in prevalence with increasing age. This was primarily driven by reduced use of COCP, while IUS use showed only a modest increase. The age-related pattern of IUS use differed from that reported for the British Armed Forces, where IUS use increased steadily with age (20). Among German servicewomen aged ≥40 years who used HC (26%), IUS represented the most frequently used method (n *=* 59, 8%), yet COCP still accounted for a substantial proportion (n *=* 55, 8%). This distribution appears to differ from current clinical gynecological guidelines, which generally recommend age-adapted contraceptive strategies and advise caution regarding COCP use in women over 40 due to thromboembolic risk (37). However factors that influence contraceptive prescribing in this age group, such as smoking status and individual cardiovascular risk profiles were not considered in the present analysis. Nevertheless, This may warrant further investigation into age-specific contraceptive counseling practices, particularly in perimenopausal servicewomen.

In any age group, HC use among German servicewomen was lower than reported for the UK (e.g. age group 18-24: 48% in Germany, 82% in the UK) (20). Further, POP use in this age group was rare in the UK (3%), whereas implants and injectable contraceptives were comparatively common. In contrast, implants and injections were virtually absent in the German cohort. Instead, POP use at 18–24 years was 10%, representing the highest proportion across all age groups in this population. Given the thromboembolic risks associated with estrogen-containing methods such as COCP (38), this pattern may reflect increased risk awareness and a critical attitude toward combined HC (32), although this assumption is speculative and cannot be confirmed with the present data. Overall, contraceptive patterns appeared to shift gradually with life-course transitions and career progression.

### 4.3 Reasons for HC use

Pregnancy prevention was the primary reason for HC use (83%), followed by the management of menstrual pain (48%) and bleeding (41%). Only a small proportion reported using HC for cycle manipulation or performance optimization, suggesting that these factors may play a minor role in contraceptive decision-making among German servicewomen. In contrast, among British female soldiers, 61% cited “stopping my periods” as a reason for taking HC, especially those using injections (20).

Among German elite athletes, current research reported that only 31% used HC for pregnancy prevention and 14% for sport-related reasons (34), suggesting that commonly assessed motives capture only part of the underlying reasons for HC use in physically active populations.

Exploratory analyses identified a small number of participants reporting the use of specific hormonal agents (e.g., Dienogest or RYEQO) primarily for the treatment of gynecological conditions such as endometriosis. More broadly, hormonal therapies may also be prescribed for the management of dysmenorrhea, heavy menstrual bleeding cycle irregularities, conditions such as polycystic ovaries or perimenopausal symptoms. Because such indications were not systematically assessed in the questionnaire, the contribution of therapeutic hormonal use to the observed prevalence of HC use may currently be underestimated. Future research should therefore aim to distinguish more clearly between contraceptive and therapeutic indications for hormonal treatment.

### 4.4 Healthcare structures and prescribing practices

Differences in HC use patterns may partly reflect variations in healthcare systems and prescribing practices across countries. In Germany, contraceptive counselling is typically individualized, without structured national programs promoting specific methods. German servicewomen receive gynecological care through the civilian healthcare system, which may further limit the integration of military-specific occupational considerations into contraceptive counseling. In the UK the introduction of an incentive framework encouraging general practitioners to counsel and prescribe LARC has contributed to a substantial increase in LARC use among British women (20). Similarly, in the United States, educational initiatives and improved access to contraceptive services have been associated with increasing uptake of long-acting methods in both civilian and military populations (15, 25).

A small proportion of servicewomen in the present study reported uncertainty regarding their contraceptive method, indicating potential gaps in knowledge and informed choice. This underscores the importance of accessible, context-specific education on women’s health and contraception within military populations. The British Armed Forces, which provide easily accessible educational resources on contraception and women’s health tailored to military demands, may serve as a model in this regard (28).

### 4.5 HC use during deployment

Operational demands appeared to have limited influence on contraceptive choice of German servicewomen. Most participants (63.8%) reported that their profession did not affect their decision, and only 10% had previously changed their contraceptive method to HC in preparation for deployment. Among those who did, depot-progestins like DMPA or implants were rarely considered (13%). These findings may reflect differences in perceived side effects, contraceptive preferences or the perceived relevance of menstrual management in operational contexts. A comparison with data from the UK shows that 43% of British servicewomen modify their contraceptive method in preparation for deployment (20).

### 4.6 Limitations and methodological considerations

This study has several limitations that should be considered when interpreting the results. First, given the cross-sectional design and reliance on self-reported data, the findings should be interpreted as descriptive associations and hypotheses-generating observations rather than evidence of causal relationships. Second, self-reported data may be subject to recall bias and social desirability bias, although participation was anonymous. Third, participation was voluntary and based on an online survey, which introduces the potential for selection bias. Consequently, the representativeness of the samples cannot be fully established. It is possible that women with a particular interest in women’s health or menstrual health were more likely to participate, whereas servicewomen with limited internet access, high operational commitments, or ongoing deployments may be underrepresented. Potentially, this has led to an underestimation of HC use, as women in austere environments might preferentially rely on methods requiring minimal logistical and hygienic effort. The direction and magnitude of this potential bias can however not be determined from the present data. The prevalence estimates reported in the present study should be interpreted as indicative rather than definite estimates for the entire population of German servicewomen. As it was not possible to determine how many servicewomen had actually received the invitation to participate, the determination of an actual response rate was limited. Nevertheless, the absolute sample size and inclusion of participants from all major service branches provide a broad overview of contraceptive practices among German servicewomen.

Furthermore, several factors known to influence contraceptive choice, including parity, pregnancy history, relationship status, educational level, and sexual activity, were not assessed and may therefore represent residual confounders when interpreting differences in contraceptive use patterns across subgroups. In addition, although selected items were based on validated instruments, the overall questionnaire was not formally validated, which may limit measurement accuracy and validity. Moreover, medical or therapeutic indications (e.g., treatment of endometriosis, dysmenorrhea or perimenopausal symptom management) were not systematically addressed. Finally, comparisons with other populations are limited by substantial heterogeneity in the categorization and reporting of contraceptive methods across studies.

### 4.7 Conclusion and future perspectives for gender-sensitive healthcare

This study provides the first comprehensive overview of HC use among German servicewomen. Overall, HC prevalence was moderate, with limited reliance on long-acting systemic progestin-only methods such as DMPA and implants. Given previously reported associations between certain systemic progestin-only methods and altersations in bone metabolism (14), the low use of these methods in the present cohort is noteworthy and may be of potential relevance in a physically demanding occupation characterized by repetitive mechanical loading. However, as bone health, injury incidence, musculoskeletal adaptations and operational readiness were not assessed in this study, the clinical implications of this finding remain unclear.

The findings may reflect that contraceptive decision-making among German servicewomen may be influenced by factors beyond operational demands alone, including broader sociocultural attitudes and healthcare structures. Independent of contraceptive practices, future preventive approaches in the German Armed Forces may therefore consider a broader range of modifiable risk factors related to musculoskeletal health, including nutrition, vitamin D status, energy availability, nicotine use, and training load management.

In addition, the findings may indicate potential gaps in military-specific education and counselling regarding contraceptive choice, occupational considerations and age-adapted contraceptive strategies. Considering the limitations discussed, future research should further explore associations between contraceptive patterns and musculoskeletal health and physical performance outcomes in German female servicemembers.

## Data Availability

The raw data supporting the conclusions of this article will be made available by the authors, without undue reservation.

## Appendix

**Appendix Table A1:** Hormonal contraceptive methods previously used during/planned for future deployments.

	Have previously changed during deployment *n* (%)	Planning to change for future deployment *n* (%)
Combined Oral Contraceptive pill (COCP)	109 (39)	98 (32)
Progesterone-only pill (POP)	53 (19)	46 (15)
Intrauterine system (IUS)	30 (11)	64 (21)
Implant	14 (5)	11 (4)
Vaginal ring	33 (12)	16 (5)
Patch	1 (0)	—
Injection	21 (7)	13 (4)
Other	8 (3)	3 (1)
Unsure	12 (4)	60 (19)

Data are n and percentage and relative to the *n =* 273 servicewomen who have indicated to have switched to a HC during a previous deployment (2. column) and the *n =* 302 servicewomen who said they are planning change to a HC in future deployments (3. column).

**Appendix Table A2:** Hormonal contraceptive use by service branch.

Service branch	n	HC prevalence n (%)	COCP n (%)	POP n (%)	Implant n (%)	Injection n (%)	IUS n (%)
Army	527	214 (41)	97 (48)	46 (23)	1 (0)	4 (2)	56 (27)
Air Force	550	211 (38)	102 (51)	47 (23)	2 (1)	2 (1)	48 (24)
Navy	335	128 (38)	61 (51)	25 (21)	0 (0)	0 (0)	34 (28)
Cyber and Information Domain	287	94 (33)	53 (58)	16 (17)	2 (2)	3 (3)	18 (20)
Medical/Support Services	782	300 (38)	147 (50)	75 (26)	6 (2)	1 (0)	64 (22)
p-value (χ²)/Cramer’s V		0.296/V = 0.045	0.225/V = 0.074				

χ²-test: HC prevalence *p =* 0.296, V = 0.045; HC type distribution *p =* 0.225, V = 0.074. Cramer’s V benchmarks: ~0.10 small, ~0.30 medium, ~0.50 large. Prevalence base: *n* per branch (HC prevalence) or HC users per branch (HC type).
